# Can Gut Microbiota Affect Dry Eye Syndrome?

**DOI:** 10.3390/ijms21228443

**Published:** 2020-11-10

**Authors:** Jayoon Moon, Chang Ho Yoon, Se Hyun Choi, Mee Kum Kim

**Affiliations:** 1 Department of Ophthalmology, College of Medicine, Seoul National University, Seoul 03080, Korea; ja-yoon88@hanmail.net (J.M.); ifree7@gmail.com (C.H.Y.); 2 Seoul Artificial Eye Center, Laboratory of Ocular Regenerative Medicine and Immunology, Seoul National University Hospital Biomedical Research Institute, Seoul 03082, Korea; choisehyun88@naver.com; 3 Department of Ophthalmology, Hallym University Sacred Heart Hospital, Anyang-si 14068, Korea

**Keywords:** dry eye, dysbiosis, gut microbiota, ocular surface, Sjögren’s syndrome

## Abstract

Using metagenomics, continuing evidence has elicited how intestinal microbiota trigger distant autoimmunity. Sjögren’s syndrome (SS) is an autoimmune disease that affects the ocular surface, with frequently unmet therapeutic needs requiring new interventions for dry eye management. Current studies also suggest the possible relation of autoimmune dry eye with gut microbiota. Herein, we review the current knowledge of how the gut microbiota interact with the immune system in homeostasis as well as its influence on rheumatic and ocular autoimmune diseases, and compare their characteristics with SS. Both rodent and human studies regarding gut microbiota in SS and environmental dry eye are explored, and the effects of prebiotics and probiotics on dry eye are discussed. Recent clinical studies have commonly observed a correlation between gut dysbiosis and clinical manifestations of SS, while environmental dry eye portrays characteristics in between normal and autoimmune. Moreover, a decrease in both the *Firmicutes*/*Bacteroidetes* ratio and genus *Faecalibacterium* have most commonly been observed in SS subjects. The presumable pathways forming the “gut dysbiosis–ocular surface–lacrimal gland axis” are introduced. This review may provide perspectives into the link between the gut microbiome and dry eye, enhance our understanding of the pathogenesis in autoimmune dry eye, and be useful in the development of future interventions.

## 1. Introduction

Microbiota are ecological communities of all microorganisms found in an environment, including bacteria, viruses, and fungi. The term “microbiome” refers to the collection of genomes from the microorganisms [1]. The impact of gut microbiota on human health has long been recognized. The NIH Human microbiome projects have provided the characterizations of the human microbiome from different body locations to determine their relations to human disease [2,3]. Among the skin, gut, and mucosal surfaces, trillions of gut microorganisms are interactive with the host cells to evolve an adaptive immune system and, sometimes, to trigger autoimmune diseases [1,4,5,6]. Since the whole genome of a free-living organism was completed in 1995 [7], the revolution in metagenome sequencing technology has enabled us to investigate the possible association between the dysbiosis of gut microbiota and human diseases [8,9,10,11]. 

Sjögren’s syndrome (SS) is a chronic autoimmune disease with dry mucosal surfaces and other systemic muscular pain [12,13]. Despite current treatment approaches for dry eye syndrome (DES) [14,15], there are still unmet needs that require the development of new interventions for SS subjects. Recent studies have revealed gut microbiota to be critical in Crohn’s disease, systemic lupus erythematosus (SLE), autoimmune uveitis, and SS-related ocular surface disease [16,17,18,19,20,21,22]. As with autoimmune diseases, metabolic diseases and the distant brain are also influenced by gut microbiota [23,24,25]. Although conjunctival or oral dysbiosis may also be involved in the pathogenesis of SS [22], this review will focus only on the gut microbiome and its relation to dry eye-associated ocular surface diseases. With an in-depth understanding of gut microbiome–immune relations in SS or non-SS dry eyes, modulation of the gut microbiota may be a promising option for future disease interventions. Herein, immune education by gut microbiota and its relation with rheumatic autoimmune diseases will be primarily introduced, followed by the current knowledge of gut dysbiosis in SS and non-SS dry eyes, and the beneficial effects of prebiotics and probiotics on DES in clinical trials and animal studies.

## 2. Innate and Adaptive Immune Education by Gut Microbiota and Homeostatic Crosstalk between Microbiota and Host

To understand gut dysbiosis-mediated diseases, it is essential to review how homeostatic crosstalk is established between the gut commensals and host immune system. The most innate and adaptive immune systems are shaped in response to gut microbial stimulation during neonatal and early periods of life. Nevertheless, through adulthood, microbial products maintain continuous crosstalk with the immune system to preserve gut homeostasis [26].

### 2.1. Innate Immune Education 

**Figure 1:**
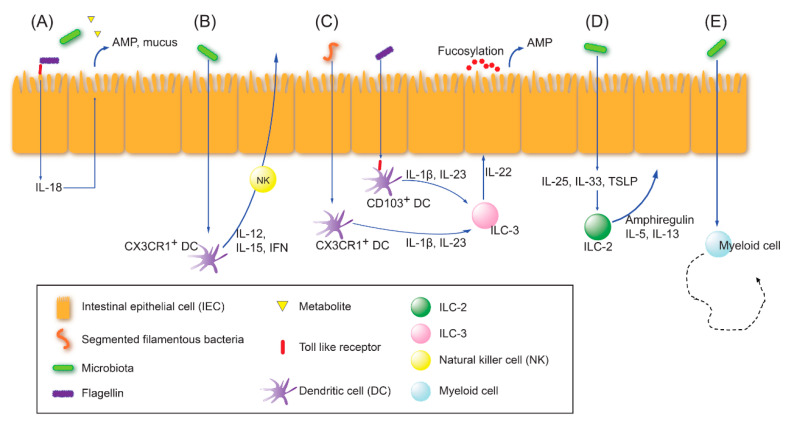
The major interplay pathways between gut microbiota and innate immune cells. (**A**) Epithelial interleukin (IL)-18 orchestrates to produce antimicrobial peptide and mucus. (**B**) CX_3_CR1^+^ dendritic cells prime natural killer (NK) cells fighting against enteric pathogens. (**C**) Group 3 innate lymphoid cells (ILC3s) produce IL-22 mediated by IL-1β and IL-23 from CD103^+^ or CX_3_CR1^+^ dendritic cells after sensing flagellin or segmented filamentous bacteria. IL-22 modulates epithelial cells to produce antimicrobial peptides and to stimulate surface fucosylation. (**D**) ILC2s produce IL-5, IL13, and amphiregulin to promote the growth of epithelial cells. (**E**) The microbiota affects the myelopoiesis in bone marrow, and the migration and phenotypes of circulating or tissue-resident myeloid cells. (Modified from the study by Thaiss et al. [27]).

The innate immune system communicates with microbiota through pattern recognition receptors (PRRs), such as Toll-like receptors (TLRs) [27]. As major players in the gut innate immune system, epithelial cells, innate lymphoid cells (ILCs), and dendritic cells sense the microbial antigens or metabolites and exert physiological responses at the host–microbiome interface [27,28]. Short-chain fatty acids (SCFAs) derived from microbiota serve as energy sources for epithelial cells and as immune modulators. There are several interplay pathways between gut microbiota and innate immune cells that protect the gut barrier (Figure 1): (1) Through the recognition of microbial products by PRRs, epithelial cells secrete interleukin 18 (IL-18), which subsequently orchestrates the production of antimicrobial peptides and mucus from epithelial cells [27]. (2) With activation by gut commensals, CX3CR1^+^ dendritic cells secrete IL-12, IL-15, and interferon (IFN) to prime natural killer (NK) cells that fight against pathogens [28]. (3) Among the ILCs (group 1, 2, and 3 ILCs), ILC3s are known to be strongly interactive with gut microbiota and produce IL-22. IL-22 production is mediated by IL-1β and IL-23 secreted from CD103^+^ or CX3CR1^+^ dendritic cells after sensing flagellin or segmented filamentous bacteria (SFB) [27,29]. IL-22 modulates epithelial cells to produce the Reg family of antimicrobial peptides (RegIIIβ and RegIIIγ) and to stimulate surface fucosylation [27,30]. IL-18- or IL-22-mediated antimicrobial peptide and fucosylated epithelial surface are able to maintain a spatial separation between the majority of enteric commensals and the epithelial layer to enhance interface barrier function [27]. (4) The microbiota may also activate ILC2s that produce IL-5, IL13, and amphiregulin to promote epithelial growth via the epithelial-derived IL-25, IL-33, and thymic stromal lymphopoietin [27,28]. (5) The microbiota have an impact on the myelopoiesis in bone marrow, and migration and phenotypes of circulating or tissue-resident myeloid cells on the mucosal surface [27]. Neutrophil aging and basophil homeostasis can be modulated by gut commensals [31,32].

### 2.2. Adaptive Immune Education 

The adaptive immune cells can recognize some specific microbial antigens, which are different from innate immune cells [26]. Naive T cells can differentiate into either effector T cells (T_eff_) to fight against pathogens, or into regulatory T cells (T_reg_) to modulate immunity depending on the type of microorganisms that they encounter. Particularly, the microbiota have an impact on B cells, T_reg_ cells, and T helper 17 cells (T_H_17), which secrete IL-17 [26]. Gut microbial exposure leads to a continuous diversification of the B-cell repertoire and production of immunoglobulin A (Ig A), and affects the balance between T_reg_ and physiologic or pathogenic T_H_17 cell responses [5,26,29]. Dysbiosis of the gut microbiota may be related with aberrant generation of autoantibodies or T_reg_/T_H_17 cell imbalance, which triggers autoimmune or metabolic diseases. 

**Figure 2:**
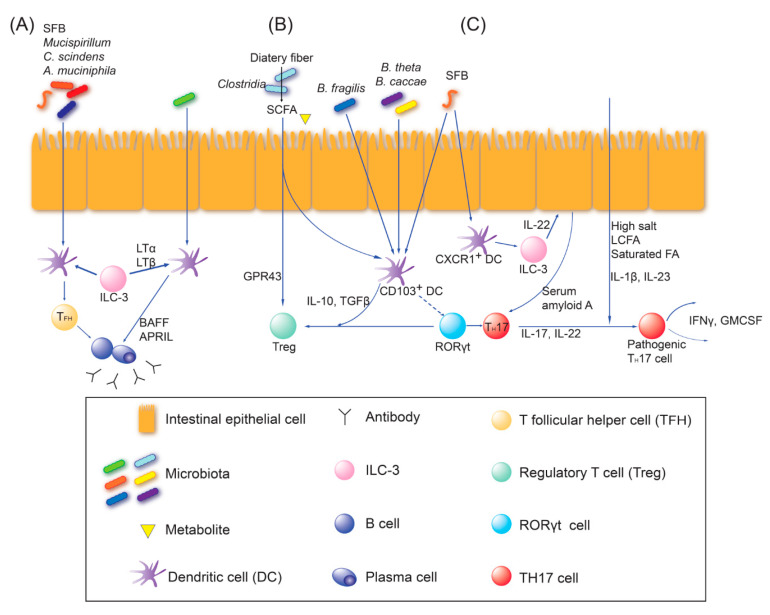
The major interplay pathways between gut microbiota and adaptive immune cells. (**A**) Ig A-producing plasma cells are activated by T follicular helper (T_FH_) cell-dependent or T_FH_ cell-independent pathways. Segmented filamentous bacteria (SFB), *Mucispirillum*, *Clostridium scindens*, and *Akkermansia muciniphila* can generate T_FH_ cell-dependent- Ig A^+^ plasma cells. Microbiota-primed group 3 innate lymphoid cells (ILC-3s) interact with dendritic cells (DCs) through Lymphotoxin (LT)α and LTβ. The activated DCs promote T_FH_ cell-independent Ig A production mediated by B-cell activating factor (BAFF) and a proliferation-inducing ligand (APRIL). (**B**) Regulatory T (T_reg_) cells can be elicited by short-chain fatty acids (SCFAs), which are produced from dietary fibers by *clusters IV*, *XIVa* and *XVIII* of *Clostridia* or by polysaccharides from certain *Bacteroides* (Phylum: *Bacteroidetes*), such as *B. fragilis, B. theta* and *B. cacae*, and *Bifidobacterium bifidum* (Phylum: *Actinobacteria*). *Lactobacillus reuteri* and *L. murinus* (Phylum: *Firmicutes*) can also induce T_reg_ cells. ILC-3s through GM-CSF, and CD103+ DCs through transforming growth factor (TGF)-β and IL-10 may interact with T_reg_ cell induction. (**C**) SFB can elicit physiologic T_H_17 cell induction whereas *Citrobacter rodentium* can induce pathogenic T_H_17 cell induction. ILC-3s and CXCR1^+^ dendritic cells facilitate induction of T_H_17 cells. Upon the abundance of IL-23 and IL-1β under the environment with higher concentrations of salt, long-chain fatty acids, and saturated fatty acids, pathogenic T_H_17 cells secrete interferon (IFN)-γ and granulocyte–macrophage colony-stimulating factor (GM-CSF). (Modified from the study by Honda and Littman [29]).

The interplay pathways between the gut microbiota and adaptive immune cells are shown in Figure 2 [5,26,29,33,34,35]. (1) Ig A-producing plasma cells are matured by T follicular helper (T_FH_) cell-dependent or T_FH_ cell-independent pathways that are mediated by epithelial or dendritic cells, and ILCs [29]. SFB, *Mucispirillum*, *Clostridium scindens*, and *Akkermansia muciniphila* are known key players that can generate T_FH_ cell-dependent plasma cells and produce Ig A. (2) Epithelial-adhering microorganisms, such as SFBs, can elicit the differentiation of naïve T cells into physiologic T_H_17 cells that produce IL-17 and IL-22, and stimulate antimicrobial peptides. Interaction between ILC3s and CXCR1^+^ or CD103^+^ dendritic cells can facilitate the induction of T_H_17 cells. T_H_17 cells induced by SFB are non-inflammatory, while T_H_17 cells elicited by *Citrobacter rodentium* secrete inflammatory cytokines [34]. Of note, SFB-induced T_H_17 cells may also become pathogenic in hosts who have a genetic predisposition to autoimmune diseases [29]. Upon abundance of IL-1β and IL-23 under an environment with higher concentrations of salt, long-chain fatty acids (LCFAs), and saturated fatty acids, T_H_17 cells become pathogenic and secrete IFN-γ and granulocyte–macrophage colony-stimulating factor (GM-CSF) [29]. When microbial-specific pathogenic T_H_17 cells move to the draining lymph nodes of target tissues, they may cross-react with self-antigens (the molecular mimicry model) or may lower the activation threshold of auto-reactive T cells (the T-cell threshold model) to trigger autoimmune diseases [29]. (3) T_reg_ cells can be elicited by SCFAs, which are produced from dietary fibers by clusters IV, XIVa, and XVIII of Clostridia or by polysaccharides from certain Bacteroides (Phylum: Bacteroidetes), such as *B. fragilis*, *B. theta* and *B. cacae*, and *Bifidobacterium bifidum* (Phylum: Actinobacteria) [26,29]. *Lactobacillus reuteri* and *L. murinus* (Phylum: Firmicutes) can also induce T_reg_ cells [11]. ILC3s under GM-CSF and CD103^+^ dendritic cells under transforming growth factor (TGF)-β and IL-10 may interact with T_reg_ cell induction. T_reg_-cell-derived IL-10 contributes to the suppression of aberrant priming of myeloid cells, γδ T, or T_H_17 cells [29]. However, how the microbial-specific T_reg_ cells exert their tolerance at mucosal surfaces or systemically still remains elusive.

## 3. Current Knowledge of the Gut Dysbiosis Associated with Non-SS Autoimmune Disease in Human Studies Detected by Metagenomic Sequencing Methods

In the healthy adult gut, *Bacteroidetes*, *Firmicutes*, *Actinobacteria*, *Proteobacteria*, and *Verrucomicrobia* are the five most abundant bacterial phyla, with the former two being the most prevalent [26]. Gut dysbiosis, seen in several autoimmune diseases, is defined as an imbalance of the gut microbiota, and is often accompanied with a disturbed or inversed *Firmicutes*/*Bacteroidetes* ratio. Significant changes of α- or β-diversities are often observed in autoimmune diseases. α-diversity is defined as variation of microbes, such as richness (i.e., number of the species) or inequality between species’ abundances in a single host, whereas β-diversity refers to the variation of microbial communities between the diseased and healthy hosts. In human and rodent studies, gut dysbiosis seems relevant to rheumatoid arthritis (RA), SLE, inflammatory bowel disease, atherosclerosis, metabolic disorders, asthma, and autism [11]. Butyrate producers, such as *Clostridia*, *Faecalibacterium*, and some species from the *Lachnospiraceae* family, which exert anti-inflammatory functions, tend to display an imbalance in autoimmune diseases.

### 3.1. Gut Dysbiosis Affects Connective Tissue Disease 

Dysbiosis of gut microbiome may be disease specific or share similar features among autoimmune diseases. Notably, primary SS (pSS) and SLE share similar characteristics in gut dysbiosis [36]. This may be related to the common key pathogenic factors, such as IFN-α, T_H_17/T_reg_ imbalance, and autoreactive B cells, that exert inflammation in RA, SLE, and pSS [37,38].

To learn whether there is a certain shared pattern between rheumatic autoimmune diseases and SS, herein, disproportional gut bacterial taxa in RA and SLE are introduced prior to discussing SS (Table 1). The dysbiotic gut microbiome pattern of SS will be discussed in the following chapters. An altered abundance of the genus *Prevotella*, *Bacteroides*, *Lactobacillus*, and *Faecalibacterium* is reported in RA subjects or an RA-like mouse model [28,39]. In particular, an expansion of *P. copri* showed a correlation to the susceptibility of arthritis [39]. Increases in *Collinsella*, *Eggerthella*, and *Faecalibacterium* were also reported in RA, and among them, the abundance of *Collinsella* showed a strong correlation with the production of IL-17A [40]. Whereas, in SLE, the abundance of *Ruminococcus gnavus* paralleled with disease activity, presumably because *R. gnavus* shares protein epitopes with the Ro60 autoantigen [41,42]. Additionally, *Bacteroides*, *Succinivibrio*, *Bilophila*, and *Parabateroides* showed a positive correlation with IL-17 and IFN-γ [43]. Conversely, *Dialister* and *Gemmiger* were negatively associated with cytokines, such as IL-17 [43]. pSS and SLE subjects shared a low *Firmicutes*/*Bacteroidetes* ratio and relatively higher abundance of *Bacteroides* species compared to healthy individuals [36].

Regarding the effects of probiotics on autoimmunity, meta-analysis of human studies showed that probiotics appear to be less effective in RA [49]. Despite the evidence on the beneficial effects of probiotics on autoimmunity in SLE-like mouse models, the preventive effects of probiotics in renal and cardiovascular complications remain controversial in human SLE [50]. Taken together, how the gut microbiota exert inflammatory functions distantly is not fully understood, and whether the modification of gut microbiota is beneficial in intervening disease should be further investigated.

### 3.2. Gut Dysbiosis Affects the Central Nervous System 

Growing evidence indicates that the brain can communicate with gut microbiota via sympathetic and vagal nerves, immune and endocrine systems, and commensal- or gut-derived neurotransmitters and neuropeptides [25,51,52,53]. Microbial-derived SCFAs either modulate the synthesis of gut-derived serotonin or circulate the blood stream to reach the brain directly and modulate microglial maturation or T_reg_ cells [52,54]. Current studies suggest that *Lactobacillus*, *Bifidobacterium*, or *Bacteroides* can ameliorate anxiety or depression and *Akkermansia muciniphila* can alleviate the symptoms of amyotrophic lateral sclerosis [51,54]. Additionally, the lack of *Dialister* and *Coprococcus* species was associated with depression [54]. A systemic review suggests that human studies with probiotics offer some benefits in major depression and Alzheimer’s disease [55].

Overall, SS and SLE share the key pathogenic features and low *Firmicutes*/*Bacteroidetes* ratio, while the other autoimmune diseases show diverse immunogenic pathways with distinct gut microbiota compositional changes.

## 4. Gut Microbiota–Immune Axis Contributes to the Pathogenesis of Non-Dry Eye Ocular Autoimmune Disease

There has been emerging evidence that gut dysbiosis contributes to the inflammatory pathogenesis of several ocular diseases [16,56,57,58,59,60,61,62,63,64]. Herein, human and animal studies are included to reveal the relation between various ocular disease and gut dysbiosis (Table 2). Through these, we can learn which characteristics of gut dysbiosis are shared between dry eye and non-dry eye ocular disease. 

### 4.1. Uveitis

Uveitis is a heterogeneous inflammatory disease of the intraocular uveal tissues [73]. An imbalance between T_H_1 or T_H_17 cells and T_reg_ cells, or antigenic mimicry with retina-specific T cells has been implicated in the pathogenesis of autoimmune uveitis [16,59,70,73]. Uveitis is attenuated by the depletion of the gut microbiota and retina-specific T cells are activated by gut commensals [59,74,75]. In autoimmune uveitis, bacteria that produce SCFAs, such as butyrate, are decreased in the gut [64,65]. A human leukocyte antigen (HLA)-B27 gene is a risk factor for ankylosing spondylitis-associated uveitis. Transgenic rats for HLA-B27/human b2-microglobulin exhibited an increased abundance of *Bacteroides vulgatus* and *Paraprevotella* [70].

Vogt–Koyanagi–Harada (VKH) is characterized by bilateral granulomatous panuveitis frequently accompanied by auditory or neurological manifestations. Likewise, T_H_1 and T_H_17 cells primed against melanocyte antigens, and/or innate immune cells activated through TLR signaling by microbial products are involved [76,77]. In VKH subjects, butyrate- or lactate-producing bacteria were depleted, and uveitis was exacerbated in recipient mice by fecal transfer from VKH subjects [57].

### 4.2. Age-Related Macular Disease (AMD)

Neovascularized AMD is characterized by retinal pigmented epithelial cell dysfunction and choroidal neovascularization [58]. High-fat diet, which accompanies gut dysbiosis, can induce retinal inflammation [58,66,71,78]. Gut neurotransmitters, such as serotonin, and microbial metabolites, are protective factors in AMD mouse models [58,79]. Gut microbiota is altered in AMD subjects [66,79]. In AMD subjects, pathobionts were enriched, whereas *Bacteroides eggerthii*, known to modulate inflammation, was decreased [66]. A high-glycemia diet increased the phylum Firmicutes and order Clostridiales that were positively associated with AMD features, whereas Bacteroidales order was negatively related with AMD features [58]. A high-fat diet also increased plasma IL-6, IL-1b, tumor necrosis factor (TNF)-α, and vascular endothelial growth factor (VEGF)-A, inducing choroidal angiogenesis [71].

### 4.3. Primary Open Angle Glaucoma (POAG)

Recent studies indicate that POAG is associated with autoimmune responses [6,56,80,81]. Gut microbiome and serum metabolites may be relevant to the pathogenesis of glaucoma [56]. In POAG subjects, proinflammatory bacteria, such as *Prevotellaceae* and *Escherichia coli,* were increased while *Megamonas* was decreased [56]. In a normal tension glaucoma mouse model, butyrate lowered the intraocular pressure [82].

### 4.4. Neuromyelitis Optica Spectrum Disorders (NMOSDs)

NMOSD is a spectrum of autoimmune demyelinating diseases characterized by myelitis and optic neuritis [67]. Anti aquaporin-4 Ig G is known to be pathogenic in NMOSD [83]. In NMOSD subjects, pathogenic genera, such as *Flavonifractor*, *Shigella*, and *Streptococcus*, were increased, whereas SCFAs-producing bacteria were depleted [67,68]. *Clostridium perfringens*, of which the epitope exhibited cross-reactivity with aquaporin-4, was increased in NMOSD subjects [69]. 

### 4.5. Inflammation of Lacrimal Gland

The lacrimal gland (LG) produces the aqueous component of tears, and various immune cells exist in the interstitial space of LG. Germ-free murine models exhibit spontaneous Sjögren-like lacrimal inflammation with reduced Ig A and Ig M production [84]. Intraperitoneal injection of *Escherichia coli* induced autoantibody production and inflammation of the Harderian glands [85]. The Ig A transcription level of LG increased in germ-free mice when *Bacteroides acidifaciens* was recolonized in the gut [72]. Lymphocytic infiltration in LG of germ-free mouse diminished after fecal transplantation [84]. Likewise, we observed that probiotics enhanced the expression of lacrimal immunomodulatory proteins in a Sjögren-like mice model [86].

In summary, the α-diversity of gut microbiota was not different in most studies. Whereas, significantly different β-diversity was consistently observed, suggesting that compositional changes of gut microbiota are associated with various ocular diseases. The *Firmicutes*/*Bacteroidetes* ratio tended to increase in EAU and AMD mouse models. *Roseburia* and *Faecalibacterium* (genus) were commonly reduced in three or more uveitis and NMOSD human studies. Moreover, gut dysbiosis correction attenuated the severity of the diseases mentioned above. Key pathogenic features, such as the imbalance in T_reg_ /T_H_17 cells and reduced SCFAs-producing commensals, are shared between uveitis and DES. Meanwhile, different pathogenic characteristics and gut dysbiosis are shown in AMD, POAG, and NMOSD. 

## 5. Pathogenesis of Non-Sjögren or Sjögren Syndrome-Related Dry Eye

The gut dysbiosis pattern is different between non-Sjögren and SS-related dry eyes. It may be involved in the pathogenesis of these diseases. Therefore, we will briefly look into their pathogenesis to discriminate these features (Table 3). 

### 5.1. Non-Sjögren Dry Eye 

DES is etiologically categorized by evaporative dry eye (EDE) and aqueous-deficient dry eye (ADDE), accompanied with tear hyperosmolarity and inflammation [15,98]. Dry eye-related oxidative stress, cellular atrophy, and senescence accelerate ocular surface damages [99,106]. Meibomian gland dysfunction (MGD), a main cause of EDE, is mediated by lid inflammation, microbial factors, hyperkeratinization, and lipid deficiencies [87,98]. Inflammatory cytokines, including IL-1, IL-6, and TNF-α, from ocular surface tissues facilitate the activation of dendritic cells [87]. Activated dendritic cells induce T_H_1 or T_H_17 cell responses, and those cells infiltrate the ocular surface and lacrimal glands [61,87]. Increased INF-γ upregulates adhesion molecules and recruits macrophages, neutrophils, and NK cells, and high IL-17 induces secretion of matrix metalloproteinases (MMPs), causing epithelial damages [87,88,89].

Considering that the gut microbiome and its products affect the expansion or imbalance of T_reg_ and T_H_17 cells [26,90,91,107,108,109], it may be associated with the pathogenesis of non-Sjögren DES. However, much less is known about the role of specific commensal microbiota on T_H_1 cell differentiation [26].

### 5.2. Sjögren Syndrome (SS)-Related Dry Eye

SS is a chronic autoimmune disease characterized by dry eyes and dry mouth due to lymphocyte infiltration of exocrine glands, including LGs [87,92]. Plasmacytoid dendritic cells (pDCs) are involved in the onset of the disease [95]. The infection of the exocrine glands by microorganisms leads to elevated type I IFN by pDCs, and to apoptosis of glandular epithelial cells, exposing self-antigens to autoantibodies, which subsequently triggers autoimmunity [95]. Both T and B cells play important roles in the pathophysiology of SS-related dry eye [12,87]. Primed T_H_1 cells produce proinflammatory cytokines (e.g., INF-γ, IL-1b, IL-6, and TNF-α), and T_H_17 cells produce IL-17. T_H_17 cells contribute to chronic inflammation and support autoreactive B cell responses [96]. CD8^+^ T cells are also involved in accelerating LG destruction [110]. Autoreactive B cells are key features of SS-related dry eyes as being pathological antigen-presenting cells (APCs) and producers of autoantibodies to Ro/SSA, La/SSB, and muscarinic 3 receptor [87,97]. These autoantibodies recruit inflammatory cells through receptor signaling or a complement activation cascade. Aquaporin (AQP)s, a family of water-permeable channels, are involved in lacrimal fluid production [103]. In SS subjects, abnormal distribution or modification of AQPs may be caused by either inflammation or prolonged hyposecretion [104]. 

Taken together, both T_H_1 and T_H_17 cells are involved in both non-Sjögren and SS-related dry eyes. Whereas, unlike non-Sjögren dry eye, autoreactive B cells and pDC-associated type I IFN are distinguished features of SS-related dry eye, similar to SLE and RA [111,112]. Given that the gut microbiota greatly affects the diversification of the B-cell repertoire with antibody production and pDCs through TLR7 signaling [26,48], gut dysbiosis is expected to be closely related to the pathogenesis of SS-related DES and will be discussed in detail in the following chapter.

## 6. Dry Eye Syndrome and Gut Microbiota

### 6.1. Evidence from Animal Studies

Along with preceding studies revealing the relationship between gut microbiota and ocular diseases, gut microbiota’s impact on dry eye has been discovered in several animal studies, especially in antibiotics-treated and germ-free animal models (Table 4). de Paiva et al. observed aggravation of desiccating stress with significant changes in the gut microbiota in C57BL/6J mice treated with antibiotics compared to those without treatment [113]. In their study, a significant decrease in goblet cell density and increased corneal barrier disruption were found. Another study by Wang et al. found a similar yet different gut dysbiosis via antibiotics treatment, where a decrease in the phyla Bacteroidetes, and increase in Proteobacteria and Firmicutes were exhibited [114]. They also observed that gut dysbiosis not only increased inflammatory cells in draining lymph nodes but also augmented the ocular surface inflammatory response to topical lipopolysaccharide administration [114]. These studies suggest that antibiotics-induced gut dysbiosis, which is depleted of commensal microbiota, is associated with increased ocular surface response to inflammation, a critical factor responsible for dry eye.

Germ-free models exhibited increased corneal barrier disruption, decreased conjunctival goblet cell density, and increased inflammatory cells, such as T_H_1 cells and IL-12^+^ dendritic cells in LGs, conjunctiva, and draining lymph nodes [84]. Wang et al. observed that after fecal transplantation, corneal barrier and goblet cell density were restored, and T_H_1 cells were decreased in the LG [84]. Similarly, Zaheer et al., found that germ-free CD25 knock-out (KO) mice exhibited increased corneal barrier disruptions, decreased conjunctival goblet cell density, and increased lymphocytic infiltrations in the LG, which were reversed with fecal transplantation [115]. In their study, antibiotics treatment also induced lymphocytic infiltration in the LG with increased secretion of IFN-γ and IL-12 [115]. These studies imply that the absence of gut commensal microbiota increases inflammation in the LG, which consequently aggravates dry eye.

Szymula et al. reported that some gut microbial peptides can activate Ro60, a major autoantigen of SS, such as *Bacterioides finegoldii*, *B*. *intestinalis*, *B*. *fragilis*, and *Alistipes finegoldii* [117]. Similarly, Yanagisawa et al. observed outer membrane protein A of *Escherichia coli* to be a stimulus of autoimmunity [85]. These studies suggest that gut dysbiosis or several specific species of gut microbiota can elicit dry eye induction or aggravation.

Recently, Wu et al. reported that, compared to standard-fat-diet mice, mice with a high-fat diet presented with profound corneal surface dysfunction with decreased tear production and goblet cell density, which may be associated with oxidative stress and induction of cellular apoptosis [116]. Though they did not investigate the gut microbiota, it can be inferred that the gut microbiota, which is greatly influenced by diet, may also be involved in dry eye aggravation [116].

It is yet uncertain whether the differences in gut microbiota results among studies are dependent on the disease pathogenesis or animal species of different genetic backgrounds. However, animal studies should be cautiously interpreted in that a finding of a specific gut bacteria in one animal study may not absolutely correlate with clinical studies.

### 6.2. Evidence from Clinical Studies

To date, there have been few studies investigating the gut microbiota of SS subjects compared to healthy individuals, and their results have been similar and yet different (Table 5). The α-diversity of the gut microbiota was reported to decrease in SS subjects [36], while others did not observe any difference [118,119]. Overall, studies agree on the presence of a significantly different gut microbiota of SS subjects compared to healthy controls through β-diversity analysis [36,113,118,119].

Clinical studies have commonly indicated that, in both DES and SS, there is a change in the composition of *Bacteroidetes* and *Firmicutes,* where *Bacteroidetes* increase while *Firmicutes* decrease, causing a decrease in the *Firmicutes*/*Bacteroidetes* ratio compared to healthy individuals [36,118,119]. Additionally, most studies have seen a decrease in the genus *Faecalibacterium* [113,119,120], while a few have seen an increased genus *Prevotella* [118,119] and decreased genus *Bifidobacterium* [118,120] and *Bacteroides* [113,119]. However, when getting closer to the bottom of classifications, a disparity in the results exists among studies. Given that gut microbiota is easily influenced by diet, ethnicity, and gender, the search for a specific causal bacteria is difficult. Still, amid these circumstances, studies have been able to find correlations between gut microbiota and clinical severity. We revealed that *Prevotella* significantly affected tear secretion while *Actinobacteria* and *Prevotella* influenced tear break up time [118]. de Paiva et al. found that gut microbiota diversity was inversely correlated with the ocular and systemic disease index of SS subjects [113]. Likewise, Mandl et al. observed that subjects with severe gut dysbiosis exhibited higher disease activity with hypocomplementemia and higher F-calprotectin [120]. Interestingly, van der Meulen et al. found that SS and SLE subjects shared a similar gut microbiota composition, which differed from healthy individuals [36]. They also noticed a positive correlation between *Clostridium sensu stricto* and serum anti-La/SSB antibody positivity [36]. Though clinical studies have difficulty in identifying a specific bacterium responsible for SS, these studies suggest an evident presence of gut dysbiosis in SS subjects compared to healthy individuals and that the degree of gut dysbiosis is correlated with clinical manifestations. Despite a common investigation of the SS, diverse patterns of gut dysbiosis are apparent among clinical studies, and so, future gut microbiota studies in SS subjects must be well aware of, and strictly control external factors. Overall, a decrease in both the *Firmicutes*/*Bacteroidetes* ratio and genus *Faecalibacterium* has been most commonly observed in SS subjects, while tendencies to increase in the *Firmicutes*/*Bacteroidetes* ratio of EAU and AMD mouse models, and a decrease in the genus *Faecalibacterium* of uveitis and NMOSD subjects were noted. 

Moon et al. observed environmental DES subjects without any autoimmune diseases to possess a gut microbiota that lies somewhere in between SS and healthy subjects, and that β-diversity revealed a significant difference from SS while none was seen when compared with healthy subjects [118]. Environmental DES subjects also exhibited a significantly decreased genus *Subdoligranulum* compared to both SS and healthy subjects [118]. Similarly, Mendez et al. also recognized a gradual change in gut microbiota composition from healthy to non-SS dry eyes, which includes environmental and other autoimmune disease-related dry eye subjects, and progressively to SS subjects [119]. These studies infer that the gut microbiota may be one of the causes for why DES occurs in some subjects while others do not.

### 6.3. Gut Microbiota Comparison of Dry Eye and Sjögren’s Syndrome

Formerly, we observed that both environmental DES and pSS subjects shared an increased genus *Veillonella* compared to healthy controls [118]. We also reported that environmental DES subjects showed a significant decrease in the genus *Subdoligranulum* compared to both SS and healthy subjects [118]. In this study, SS subjects exhibited significantly different gut microbiota compared to both DES and healthy controls while DES and healthy controls revealed no difference upon β-diversity analysis [118]. On the other hand, Mendez et al. reported that non-SS subjects with DES and possibly other overlapping autoimmune diseases, and SS subjects had similar gut microbiota [119]. This disparate result may be due to the different inclusion criteria of DES or non-SS in each study, which was seen in a previous study that SS and SLE share similar gut dysbiosis features [36]. However, both studies agreed on a gradual change of gut microbiota from healthy to diseased subjects [118,119]. Moreover, studies noticed that several gut bacteria were associated with the severity of dry eye parameters [113,118,119]. While SS and DES subjects’ gut microbiota exhibit a couple of discriminating characteristics from healthy controls and each other, studies show that these distinguishing features are connected, creating a spectrum that gradually shifts from healthy to DES to SS. Therefore, it is presumed that depending on where a subject’s gut microbiota lies along this gut microbiota spectrum, the severity of clinical manifestations is determined and consequently whether it expands further to autoimmune diseases, such as SS, is decided.

## 7. Dry Eye and Probiotics

### 7.1. Prebiotics and Probiotics

Probiotics are defined as live microorganisms that present health benefits when administered in adequate amounts [121]. Prebiotics refer to substrates that microorganisms use to bestow health benefits upon the host [122]. Both probiotics and prebiotics have received much spotlight over the past decade for their advantages in coordinating gut microbiota to help ameliorate several diseases [123]. While several studies have obtained beneficial effects in several autoimmune diseases [123,124], evidence regarding their effects on the ocular surface, especially dry eye, is now just emerging (Table 6).

### 7.2. Effects Seen in Animal Studies

NOD.B10.H2*^b^* (NOD) mice treated with prebiotic xylooligosaccharides resulted in reduced sialadenitis and insulitis by increasing regulatory macrophages and activating T_reg_ cells while lowering cytotoxic T cells [130]. Interestingly, this study also observed that a combination with antibiotics increased the clinical benefits of prebiotics regarding insulitis but not sialadenitis [130], which implies that each species of gut microbiota affects each target organ in a different manner. On the other hand, recent animal studies regarding dry eye and probiotics have commonly observed that while antibiotics treatment increases dry eye, prebiotics and probiotics induce clinical benefits with mitigation of inflammatory cells (Table 6). Kawashima et al. observed that *E. faecium* WB2000 mixed with fish oil increased tear secretion and decreased reactive oxygen species production in LGs of desiccating-stressed rats [125]. Two studies have observed that a probiotic composed of *L. casei*, *L*. *acidophilus*, *L. reuteri*, *B. bifidum*, and *S. thermophiles* for 3 weeks in NOD mice restored corneal barrier disruption and increased tear secretion [86,126]. We noticed a decrease in inflammatory cell infiltration in LG and CD8^+^ IFN-γ**^Hi^** cells in the lymph nodes, while T_reg_ cells increased [126]. While using the same probiotic in the same SS model, Choi et al. observed that proteins related with antigen presentation decreased in the LGs [86]. 

These animal studies indicate that probiotics and prebiotics can affect the gut microbiota and carry out variable clinical and immunological changes. Given that T and B cells are the main source of the mechanism in SS subjects while T cells are more dominant in environmental DES, probiotics’ and prebiotics’ effects on the gut microbiota and subsequently to clinical and immunological manifestations may differ according to the type of studied animal model. These possible differences among studied animals should be considered in future animal studies.

### 7.3. Effects Seen in Clinical Studies

Clinical benefits from probiotics on dry eyes have been investigated in a few human studies (Table 6). Though *E. faecium* is known for being an opportunistic pathogen, some of its strains are validated to be safely used as probiotics [131]. Some strains possess pathways to enable the production of essential amino acids and vitamins, which are important in human health [131]. Likewise, Kawashima et al. observed that intake of *E. faecium* WB2000 mixed with fish oil for 8 weeks alleviated subjective symptoms with increased tear secretion in DES subjects [125]. Similarly, a mixture of *E. faecium* LMG S-28935 and *Saccharomyces boulardii* MUCL 53837 decreased subjective symptoms with an increase in both tear secretion and tear break-up time [127]. *Saccharomyces* is also a well-known SCFAs-producing bacteria [132]. Lactobacillus and Bifidobacterium, renowned for their many species associated with lactic acid and acetic acid production, are regarded as the main ingredient for various probiotics [133]. A pilot study by Chisari et al. reported that a 30-day supplementation of *B. lactis* and *B. bifido* significantly increased tear secretion and tear break-up time in 20 DES subjects compared to placebo [128]. Additionally, a processed H_2_-producing milk, as a prebiotic supplement, exhibited similar clinical effects in DES subjects [129]. Despite these positive clinical results, the safety of probiotics use in immunocompromised SS subjects is warranted, where administration of *Lactobacillus* spp. was reported to possibly act as an opportunistic pathogen [134]. However, overall, clinical studies have observed probiotics to be safe and to not only alleviate subjective symptoms but also increase both tear secretion and tear break-up time. These clinical results suggest the advantages of diverse probiotics as a supplementary treatment to DES. Therefore, future clinical studies concerning SS subjects are now necessary to further elucidate and expand probiotics’ benefits. 

## 8. The Hypothesis of Gut Dysbiosis–Ocular Surface–Lacrimal Gland Axis Communications

**Figure 3:**
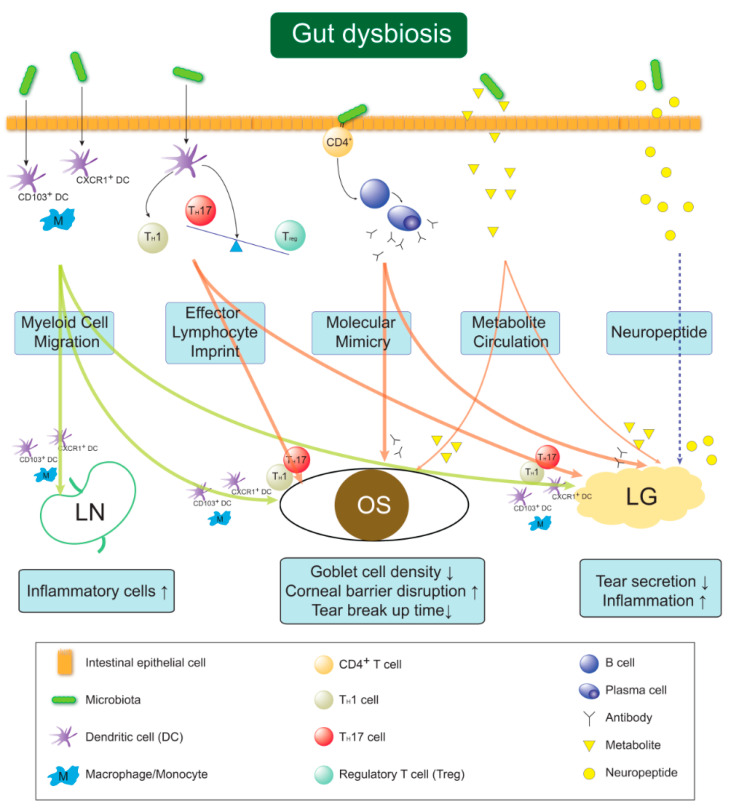
The hypothesis of Gut dysbiosis–Ocular surface–Lacrimal gland Axis. Gut dysbiosis may induce dry eye disease by the following five mechanisms. Myeloid cell migration theory; Gut dysbiosis-mediated CD103^+^ or CXCR1^+^ dendritic cells or monocyte/macrophages migrate to drainage lymph nodes, ocular surface and lacrimal glands in order to prime T cells or secrete pro-inflammatory cytokines. Effector lymphocyte imprint theory; Gut-derived helper T 1 (T_H_1) and 17 (T_H_17) cells migrate to the ocular surface and lacrimal gland, or gut-derived T_reg_ cells are less circulated. Molecular mimicry theory; Microbial-derived antigens cross-prime autoreactive CD4^+^ T cells helping B cells to produce autoantibodies. Metabolite circulation theory; Microbial metabolites, such as short-chain fatty acids, decrease to enter systemic circulation reaching ocular surface and lacrimal gland. Neuropeptide circulation theory; Homeostatic circulation of gut-derived neuropeptides is distributed to reach lacrimal gland and influence tear secretion.

Key pathogenetic factors of DES are tear hyperosmolarity or inflammatory cascades, wherein T_H_1, T_H_17/T_reg_ imbalance, NK cells, or monocytes serve as culprits on the ocular surface, and also autoreactive B cells in SS [37,87,92,135]. Given that the gut microbiota affects these cells and their related cytokines, DES is possibly initiated or aggravated through the crosstalk of the “gut microbiome–ocular surface–lacrimal gland” axis [22]. Both antibiotics-treated and germ-free murine models have exerted ocular surface and lacrimal gland inflammations [84,113,114,115]. Fecal transplantations reversed these distinctive dry eye features [84,115]. These findings emphasize gut dysbiosis’ contribution to the pathogenesis of autoimmune DES. Herein, presumable communication routes creating the “gut dysbiosis–ocular surface–lacrimal gland axis” are presented in autoimmune dry eye (Figure 3).

First, activated dendritic cells or monocyte/macrophages mediated by gut dysbiosis migrate to the drainage lymph node and ocular surface to prime naïve T cells into T_eff_ cells or to secrete proinflammatory cytokines in the ocular surface and LG (myeloid cell migration theory). Second, either gut-primed T_H_1, T_H_17 cells, or autoreactive B cell-derived immunoglobulins migrate directly to the ocular surface and LG to exert inflammation, or the reduced circulating population of gut-derived T_reg_ subsequently increases the inflammation of the ocular surface and LG (effector lymphocyte imprint theory). Third, microbial-derived antigens that possess similar epitopes, such as Ro/SSA autoantigen, cross-prime autoreactive T and B cells, which consequently produce anti-Ro/SSA autoantibodies to initiate SS [85,117] (molecular mimicry theory). Fourth, gut dysbiosis-derived SCFA reduction is associated with autoimmune diseases [136]. In the former chapters, we observed a decrease in the genus *Faecalibacterium,* one of the main SFCA-producing bacteria, in SS subjects [113,119,120] and so it can be inferred that a reduction in SFCA influences a decrease in tear secretion. Likewise, a decrease in SCFA affect distant autoreactive T cells of the ocular surface and LG (metabolite circulation theory). Finally, given that neuropeptides, such as neuropeptide Y, substance P, vasoactive intestinal polypeptide, and calcitonin gene-related peptide, have been observed in the gut–brain axis [53,137] and the critical roles of several neuropeptides, including the above mentioned, taken place in stimulating LG tear secretion [87], the disturbances of gut-derived neuropeptides can control LG secretion. Therefore, this is considered as the last hidden mechanism for increased tear secretion seen in animal and human studies with pre- and probiotics administration [86,125,126,127,128,129] (neuropeptide circulation theory).

## 9. Conclusions and Perspectives

Since the advancement of metagenomic sequencing has enabled a new level of perspective on human microbiome, the impact of gut microbiota on human health and autoimmune diseases has long been acknowledged. Whereas, the beneficial or harmful effects of the gut microbiome in the pathogenesis of dry eye and other autoimmune ocular diseases are now just beginning to be understood. 

The gut innate immune system that includes gut epithelial cells, ILCs, and dendritic cells exerts protective responses through key cytokines, such as IL-18 and IL-22, and antimicrobial peptides at the host–microbiome interface. Gut dysbiosis leads to an aberrant diversification of the B-cell repertoire and an imbalance between T_reg_ and T_H_17 cell responses in adaptive immunity, subsequently triggering ocular autoimmune diseases. Both non-Sjögren and SS-related dry eyes as well as uveitis share key pathogenic features, such as an imbalance in T_reg_ /T_H_17 cells, or reduced SCFAs-producing bacteria. Whereas, activation of autoreactive B cells and pDCs is a distinguished characteristic of SS-related DES and SLE compared to non-Sjögren DES. Although the Sjögren-like rodent models show similar pathogenic features to humans, the compositions of gut dysbiosis are clearly distinct from those of human SS. Therefore, careful interpretation and application of animal studies of specific gut bacteria to clinical human studies are warranted.

Current human studies have commonly observed a correlation between gut dysbiosis and clinical manifestations of SS, while environmental dry eye places its characteristics in between normal and SS. Of note, SS subjects, from most studies have possessed a decrease in both the *Firmicutes/Bacteroidetes* ratio and genus *Faecalibacterium*. A reduced genus *Faecalibacterium* has also often been seen in both uveitis and NMOSD subjects. This indicates that the distinct gut dysbiosis affecting autoimmune dry eye can also sometimes possess overlapping gut dysbiotic features in other diseases. The outcomes of human studies suggest the advantages of probiotics and prebiotics in the management of DES. However, functional studies of gut microbiota are still preliminary to fully understand the pathogenesis of dry eye associated with gut dysbiosis. Therefore, mechanical investigations are now necessary to further elucidate key communication routes of the “gut dysbiosis–ocular surface–lacrimal gland axis” and to establish customized interventions with an optimized modulation of the gut microbiota to treat dry eye. 

## Figures and Tables

**Table 1:** Human studies of rheumatoid arthritis and systemic lupus erythematosus showing gut dysbiosis detected by metagenomic methods.

Author, Year	Disease	Class	Increased	Decreased	(+/−) Disease Correlation
Scher, 2013 [39]	RA	Species	*P. copri*		(+) *P. copri*
		Genus	*Prevotella*	*Bacteroides*	(+) *Prevotella*
Chen, 2016 [40]		Genus	*Collinsella, Eggerthella, Faecalibacterium*		(+) *Collinsella*
Vaahtovuo, 2008 [44]		Genus /species		* Bacteroides, Bifidobacteria Porphyromonas, Prevotella, /B. fragilis, C. coccoides E. rectale, *	
He, 2016 [45]	SLE	Genus	*Eggerthella, Eubacterium, Flavonifractor, Incertae sedis, Klebsiella, Prevotella, Rhodococcus*	*Dialister, Pseudobutyrivibrio*	
Hevia, 2014 [46]		Genus	*Bacteroides spp*		
Guo, 2020 [43]		Genus	*Bacteroides, Bilophila, Coprococcus, Parabacteroides, Prevotella, Succinivibrio*	*Dialister, Gemmiger*	(+) *Bacteroides, Succinivibrio,* *Bilophila, Parabateroides* (−) *Gemmiger, Dialister*
Luo, 2018 [47]		Genus	*Blautia*	*Odoribacter*	
van der Meulen, 2019 [36]		Genus	*Alistipes, Bacteroides, Proteobacteria*		
		Species	*B.ovatus, B. theta, B. uniformis, B. vulgatus*		
Zegarra-Ruiz, 2019 [48]		Genus	*Lactobacillus*		
Azzouz, 2019 [42]		Species	*Ruminococcus gnavus*		(+) *Ruminococcus gnavus*

RA; Rheumatoid arthritis, SLE; Systemic lupus erythematosus; (+): positive correlation with disease severity, (−): inverse correlation with disease severity.

**Table 2:** Representative gut dysbiosis in various ocular diseases of the human and mouse.

Author, Year	Analysis	Diversity	Taxa			Interpretation
			Classes	Increased	Decreased	
Human study						
Kalyana, 2018 [65]	Uveitis	α↓ β+	Family		*Lachnospiraceae, Ruminococcaceae*	↓Anti-inflammatory bacteria ↓Butyrate producing bacteria ↑Proinflammatory bacteria
			Genus		*Bacteroides, Blautia, Clostridium, Coprococcus, Dialister, Dorea, **Faecalibacterium**, Lachnospira, Odoribacter, Oscillospira, Megasphaera, Mitsuokella,* ***Roseburia*** *, Ruminococcus*	
			Species	*Prevotella copri*	*Akkermansia municiphala, Bifidobacterium adolescentis, **Faecalibacterium** prausnitzii, Veillonella dispar*	
Huang, 2018 [60]	Uveitis	α→ β+	Class	*Negativicutes*		(+) *Roseburia* ∝ linoleic acid
			Order	*Oceanospirillales, Selenomonadales*		
			Family	*Clostridiales_Incertae Sedis XI, Halomonadaceae*		
			Genus	*Veillonella*	***Roseburia***	
Ye, 2020 [57] ^a^	VKH	α→ β-	Genus		*Azospirillum*	↓Butyrate or lactate producing bacteria ↑Gram(−) bacteria
			Species	*Paraprevotella clara*	*Azospirillum spp., Bifidobacterium spp., Clostridium spp.*	
Ye, 2018 [61] ^a^	Behcet’s disease	α: NA β: NA	Phylum	* Actinobacteria, Proteobacteria *		↓Butyrate producing bacteria ↑Opportunistic bacteria
			Species	* Actinomyces spp., Bilophila spp.*, *Corynebacterium spp.*, *Fusobacterium spp.*, *Parabacteroides spp. *, * Paraprevotella spp. *, * Stenotrophomonas spp. *	* Clostridium * * spp. *	
Shimizu, 2019 [62]	Behcet’s disease	α→ β+	Species	*Acidaminococcus spp., Bifidobacterium bifidum, Eggerthella lenta, Lactobacillus iners, Lactobacillus mucosae, Lactobacillus salivarius, Streptococcus spp.*	* Butyrivibrio species, Filifactor species, Streptococcus infantis *, * Megamonas hypermegale *	↓SCFAs producing bacteria
Zinkernagel, 2017 [66] ^a^	NAMD	α: NA β+	Family	*Oscillospiraceae*		↑ Inflammatory bacteria
			Genus	*Anaerotruncus*		
			Species	*Eubacterium ventriosum, Ruminococcus torques*	*Bacteroides eggerthii*	
Gong, 2020 [56]	POAG	α→ β+	Family	*Enterobacteriaceae, Prevotellaceae*		(−) ***Faecalibacterium*** ∝ VF-MD (+) *Streptococcus* ∝ RNFLT
			Genus		*Megamonas*	
			Species	*Escherichia coli*	*Bacteroides plebeius*	
Shi, 2020 [67]	NMOSD	α→ β+	Genus	*Flavonifractor, Streptococcus*	*Blautia, Coprococcus, **Faecalibacterium**, Fusicatenibacter Lachnospiracea_incertae_sedis, Prevotella, Romboutsia, **Roseburia**,*	↑Pathogenic bacteria ↓Commensal bacteria
Gong, 2019 [68]	NMOSD	α→ β+	Genus	*Shigella, Streptococcus*	***Faecalibacterium*** *,* *Lachnospira **Roseburia***	↓SCFAs producing bacteria ↑Pathogenic bacteria (+) *Streptococcus* disease severity
			Species	*Streptococcus* *spp. (S. oralis, S. salivarius, S. parasanguinis, S. pneumonia, and S. mitis)*		
Cree, 2016 [69]	NMOSD	α: NA β+	Species	*Clostridium perfringens*		
Mouse study						
Janowitz, 2019 [63]	EAU	α↓ β+	Phylum	***Firmicutes***	***Bacteroidetes*** *, Proteobacteria*	↑F/B ratio (−) α-diversity ∝ uveitis
			Class		*Bacteroidia*	
			Genus	*Anaeroplasma, Clostridium, Lactobacillus, Parabacteroides, Prevotella*	*Desulfovibrio, Ruminococcus*	
Du, 2020 [64]	EAU ^b^	α→ β+	Class	*Bacilli*		
			Family	*Anaeroplasmataceae, Lactobacillaceae*	*Muribaculaceae, Ruminococcaceae*	↑*Lactobacillaceae,* increasing type I IFN ↓T_reg_-enhancing Bacteria
			Order	*Anaeroplasmatales, Lactobacillales*		
			Genus	*Anaeroplasma, Lactobacillus*	*Akkermansia, Bacteroides,* *Oscillibacter*	
			Species	*Lactobacillus gasseri, Lactobacillus intestinalis*	*Bacteroides sartorii, Parabacteroides goldsteinii*	
Lin, 2014 [70]	AS	α: NA β+	Genus	*Paraprevotella*		↑RA-causing Bacteria
			Species	*Bacteroides vulgatus*	*Akkermansia muciniphila*	
Andriessen, 2016 [71]	AMD	α→ β+	Phylum	***Firmicutes*** *,* *Proteobacteria*	***Bacteroidetes***	↑F/B ratio
Rowan, 2017 [58]	AMD	α↑ β+	Phylum	***Firmicutes***	***Bacteroidetes***	↑F/B ratio (+) *Firmicutes, Clostridia* ∝ phenotype (−) *Bacteroidales, Erysipelotrichi* ∝ phenotype
			Class	*Clostridia*	*Erysipelotrichi*	
			Order		*Bacteroidales*	
Kugadas, 2017 [72]	SW vs. B6	α↓ β: NA	Genus	*Bacteroides,* *Dysgonomonas, Prevotella*		(+) *Bacteroides acidifaciens* ∝ LG IgA transcription

^a^ Studies analyzed using whole-genome shotgun sequencing (3 studies), otherwise studies using 16s rRNA sequencing. ^b^ The EAU group was compared to the EAU group treated with berberine. **Bold font** indicates common microbiota findings in 3 or more studies. AMD, Age-Related Macular Degeneration; AS, ankylosing spondylitis; B6, C57BL6/N; EAU, Experimental autoimmune uveitis; F/B ratio, *Firmicutes/Bacteroidetes* ratio; IFN, interferon; LG, lacrimal gland; NA, not available; NAMD, Neovascular AMD; NMOSD, Neuromyelitis optica spectrum disorders; RA, rheumatoid arthritis; T_reg_, regulatory T cell; RNFLT, retinal nerve fiber layer thickness; SCFA, short-chain fatty acid; SPF, specific pathogen-free; SW, Swiss Webster; sp., species; VF-MD, visual field mean defect; VKH, Vogt–Koyanagi–Harada disease; vs., versus; →, no difference compared to control; +: significant difference compared to control; ↓, decreased compared to control; ↑, increased compared to control. (+): positive correlation, (−): inverse correlation, A ∝ B: correlation between A and B

**Table 3:** Pathogenesis of non-Sjögren or Sjögren’s syndrome-related dry eye.

	Non- Sjögren Dry Eye	Sjögren’s Syndrome-Related Dry Eye
Immunologic	IL-1↑, IL-6↑,TNF-α↑ MMPs↑ Neutrophil↑, NK cell↑, Macrophage↑ T_H_1/ IFN-γ↑ T_H_17/IL-17↑ [87,88,89,90,91,92,93,94]	Type I IFN (α, β)↑ TNF-α↑, IL-2, IL-7↑ BAFF↑ T_H_1/ IFN-γ↑, T_H_17/ IL-17↑ Cytotoxic CD8^+^ T cell↑ B cell↑, Autoantibody↑ pDC in gland↑ [12,87,95,96,97]
Non-immunologic	Hyperkeratinisation of MG orifice Atrophy Oxidative stress Senescence [87,98,99,100,101,102]	Hormone AQP dysfunction? [87,103,104,105]

AQP, aquaporin; BAFF, B-cell-activating factor; DC, dendritic cell; IFN, interferon; IL, interleukin; MG, meibomian gland; MMPs, matrix metalloproteinases; NK, natural killer; pDC, plasmacytoid DC; TH1, T helper type 1; TH17, T helper type 17; TNF, tumor necrosis factor.

**Table 4:** Dry eye rodent models related with gut microbiota.

Author, Year	Treatment	Subjects	Representative Gut Microbiota	Change in OS/LG/dLN
de Paiva, 2016 [113]	Antibiotics ^a^ (24 days)	DS B6	Number of OTUs↓ α↓ β+ ↓ *Alistipes, Allobaculum, Bacteroides, Blautia, Clostridium, Desulfovibrio, Intestinimonas, Lactobacillus* (Genera) ↑ *Enterobacter, Escherichia/Shigella, Parasutterella, Pseudomonas, Staphylococcus* (Genera)	Goblet cell density↓ Corneal staining↑ CD4^+^ T cell↑(OS) IFN-γ↑(OS) IL-13↓(OS)
Wang, 2018 [84]	Germ-free	B6		Goblet cell density↓ Corneal staining↑ Tear EGF↓ CD8^+^ & CD4^+^ T cell↑(LG) T_H_1^+^ cell↑(LG) IL-12^+^CD11b^+^CD11c^+^ cell↑(OS, dLN) MHC II, IFN-γ, IL-12 & Caspase 3↑(LG)
Zaheer, 2018 [115]	Germ-free	CD25KO		Goblet cell density↓ Corneal staining↑ T_H_1^+^ cell↑(LG, dLN) B220^+^ cell↑(LG) IL-12^+^CD11c^+^MHC^−-^ cell↑(LG, dLN, OS) IFN-γ & IL-12↑(LG)
	Antibiotics ^a^ (28 days)	CD25KO	N/A	IFN-γ & IL-12↑(LG)
Wang, 2019 [114]	Antibiotics ^a^ (14 days)	B6	α↓ β+ ↓*Akkermansia, Bacteroidales, Bacteroides, Lachnospiraceae, Oscillibacter, Parasutterella, Ruminococcaceae, Ruminiclostridium* (Genera) ↑*Bacillus,* *Curtobacterium,* *Escherichia/Shigella, Firmicutes,* *Lactococcus, Megasphaera, Staphylococcus* (Genera)	Serum LPS↑ MHC II^+^CD11c^+^CD11b^+^ cell↑(dLN) Inflammatory response ^b^ to topical LPS↑(cornea)
	Germ-free	B6		Inflammatory response ^c^ to topical LPS↑(OS)
Wu, 2020 [116]	High-fat diet ^d^	B6	N/A	Goblet cell density↓ Corneal staining↑ Tear secretion↓ Squamous metaplasia↑(OS) Oxidative stress/apoptosis↑(OS)

^a^ Ampicillin, Gentamicin, Metronidazole, Neomycin, Vancomycin; ^b^ TNF-α, CXCL10 & IL-12 mRNA by RT-PCR; ^c^ CXCL10, IL-12 & IFN-γ mRNA by RT-PCR; ^d^ 60 kcal% fat diet (standard fat diet = 10 kcal% fat diet); OS, ocular surface; LG, lacrimal gland; dLN, draining lymph node; DS, desiccating stressed; B6, C57BL/6J mice; N/A, not available; EGF, epidermal growth factor; APC, antigen presenting cells; CD25KO, CD25 knock-out; LPS, lipopolysaccharide; vs, versus; →, no difference compared to control; +: significant difference compared to control; ↓, decreased compared to control; ↑, increased compared to control.

**Table 5:** Human studies of dry eye-related representative gut microbiota.

Author, Year	α	Class	Increased	Decreased	(+/−) Disease Correlation
pSS					
de Paiva, 2016 [113]	↓	Genus	*Anaerostipes, Bifidobacterium, Bilophila, Blautia, Escherichia/Shigella, Lachnospira, Moryella, Pseudobutyrivibrio, Streptococcus*	*Bacteroides, **Faecalibacterium**, Haemophilus, Odoribacter, Parabacteroides, Prevotella*	(−) Diversity ∝ ocular/systemic disease index
Mandl, 2017 [120]	N/A	Genus	N/A	*Alistipes, Bifidobacterium*	(+) Dysbiosis ^a^ ∝ disease activity /F-calprotectin (−) Dysbiosis ^a^ ∝ complementemia
		Species	N/A	***Faecalibacterium prausnitzii***	
van der Meulen, 2019 [36]	↓	Phylum	***Bacteroidetes*** *, Proteobacteria*	***Firmicutes*/*Bacteroidetes* ratio**	(+) *Clostridium sensu stricto* ∝ anti-La/SSB antibody
		Genus	*Alistipes, Bacteroides, Barnesiella, Lachnosclostridium, Lachnospira, Parasutterella*	*Actinomyces, Clostridium sensu stricto, Enterorhabdus, Romboutsia, Senegalimassilia, Slackia, Turicibacter*	
		Species	*Bacteroides ovatus, Bacteroides uniformis, Bacteroides vulgatus*		
Moon, 2020 [118]	-	Phylum	***Bacteriodetes***	***Firmicutes*/*Bacteroidetes* ratio**, *Actinobacteria*	^b^ (−) *Prevotella* ∝ tear secretion (+) *Actinobacteria* ∝ TBUT (−) *Prevotella* ∝ TBUT
		Class		*Clostridia*	
		Genus	*Alistipes, Odoribacter, Prevotella, Veillonella*	*Agathobacter, Bifidobacterium, Blautia, Dorea*	
		Species		*Bifidobacterium longum, Eubacterium hallii*	
DES ^c^					
Moon, 2020 [118]	-	Genus	*Veillonella*	*Subdoligranulum*	^b^
pSS & non-SS ^d^					
Mendez, 2020 [119]	-	Phylum	*Actinobacteria, **Bacteroidetes**, Proteobacteria*	***Firmicutes***	(+) *Eubacteriaceae, Eggerthellaceae*, ∝ DEQ5 (−) *Ruminococcaceae* ∝ DEQ5 (−) *Akkermanciaceae* ∝ tear secretion
		Order		*Clostridiales*	
		Family	*Actinomycetaceae, Akkermanciaceae, Coriobacteriaceae, Eggerthellaceae, Eubacteriaceae, Lactobacillaceae*	*Lachnospiraceae, Ruminococcaceae*	
		Genus	*Megasphaera, Parabacteroides, Prevotella*	*Bacteroides, Faecalibacterium, Veillonella*	

^a^ Dysbiosis index score defined by GA-map™ Dysbiosis Test (Genetic Analysis, Oslo, Norway), ranging from 1 to 5; ^b^ Results are from univariate and multivariate linear regression analysis that applied healthy, dry eye syndrome and primary Sjögren’s syndrome subjects altogether.; ^c^ Defined as subjects with dry eye symptoms and tear break up time < 10 seconds; ^d^ Defined as subjects that do not fully meet the 2016 American College of Rheumatology criteria for pSS and has dry eye symptoms; **Bold font** indicates common microbiota finding among half or more studies; pSS, primary Sjögren’s syndrome; SLE, systemic lupus erythematosus; DES, dry eye syndrome; N/A, not available; DEQ5, dry eye questionnaire 5; -: no significant difference from control, (+): positive correlation, (−): inverse correlation; A ∝ B: correlation between A and B.

**Table 6:** Effects of probiotics or prebiotics on dry eye in rodent and human studies.

Author, Year	Tx	Tx Period	Subjects	Representative Gut Microbiota	Change in OS/LG/dLN
Rodent Study					
Kawashima, 2016 [125]	Fish oil, lactoferrin, zinc, vitamin C, lutein, vitamin E, γ-aminobutanoic acid & *E. faecium WB2000*	2 days	DS rats	N/A	Tear secretion↑ ROS↓(LG)
Kim, 2017 [126]	*L. casei, L. acidophilus, L. reuteri, B. bifidum* & *S. thermophiles*	3 weeks	NOD.B10.*H2 ^b^*	N/A	Tear secretion↑ Corneal staining↓ Inflammation foci ^a^↓(LG) CD8^+^IFN-γ^hi^ T cell↓(dLN) T_reg_ cell↑(dLN)
Choi, 2020 [86]	*L. casei, L. acidophilus, L. reuteri, B. bifidum* & *S. thermophiles*	3 weeks	NOD.B10.*H2 ^b^*	↑*Lactobacillus helveticus, L. hamsteri, L. reuteri, L. casei, L. brantae, L. amylovorous, Akkermansia municipila, Aerococcus viridans, B. bifidum, Streptococcus salivarius* ↓*Lactobacillus intestinalis*	Tear secretion↑ Corneal staining↓ Immune response genes ^b^↓(LG) IL-10↑(OS) IL-1b↓(OS)
Human Study					
Kawashima, 2016 [125]	Fish oil, lactoferrin, zinc, vitamin C, lutein, vitamin E, γ-aminobutanoic acid & *E. faecium WB2000*	8 weeks	DES ^c^	N/A	Scored subjective symptoms ^d^↓ Tear secretion↑
Chisari, 2017 [127]	*S. boulardii MUCL 53837* & *E. faecium LMG S-28935*	30 days	DES ^e^	N/A	Subjective dry eye symptoms ^f^↓ TBUT↑ Tear secretion↑
Chisari, 2017 [128]	*B. lactis DSM 25566* & *B. bifido DSM 25565*	30 days	DES ^e^	N/A	Tear secretion↑ TBUT↑
Kawashima, 2019 [129]	Hydrogen-producing milk	3 weeks	DES ^c^	N/A	TBUT↑ (♀)

^a^ Inflammatory foci score; >50 inflammatory cells/focus = 1, 25–50 inflammatory cells/focus = 0.5; ^b^ Ptprc, Hmgb2, Psmb8, H2-Aa, H2-K1, Psme1, Tap1, Tap2 & Psmb9; ^c^ Subjects with dry eye symptoms, qualitative or quantitative disturbance of the tear film (Schirmer test ≤ 5 mm or TBUT ≤ 5 s) and total fluorescein staining score of at least 3 points.; ^d^ Total score, foreign body sensation, dry eye sensation and ocular fatigue (evaluated by Dry Eye-Related Quality-of-Life Score); ^e^ Subjects defined to have dry eye syndrome clinically or pathologically; ^f^ Dry eye symptom severity, frequency of pain or soreness in ocular fatigue, eyelid heaviness, eye redness and foreign body sensation (evaluated by Ocular Surface Disease Index); Tx, treatment; OS, ocular surface; LG, lacrimal gland; dLN, draining lymph node; DS, desiccating stressed; B6, C57BL/6J mice; ROS, reactive oxygen species; DES, dry eye syndrome; TBUT, tear break up time; ♀, female; 8-OHdG, 8-hydroxydeoxyguanosine.

## Abbreviations

ADDE	Aqueous deficient dry eye
AMD	Age related macular disease
APC	Antigen-presenting cell
AQP	Aquaporin
DES	Dry eye syndrome
EDE	Evaporative dry eye
GM-CSF	Granulocyte–macrophage colony-stimulating factor
HLA	Human leukocyte antigen
IFN	Interferon
Ig	Immunoglobulin
IL	Interleukin
ILC	Innate lymphoid cell
KO	Knock-out
LCFA	Long-chain fatty acid
LG	Lacrimal gland
MGD	Meibomian gland dysfunction
MMP	Matrix metalloproteinase
NK	Natural killer
NMOSD	Neuromyelitis optica spectrum disorders
NOD	NOD.B10.H2^b^
pDC	Plasmacytoid dendritic cell
POAG	Primary open angle glaucoma
PRR	Pattern recognition receptor
pSS	Primary Sjögren’s syndrome
RA	Rheumatoid arthritis
SFB	Segmented filamentous bacteria
SFCA	Short-chain fatty acid
SLE	Systemic lupus erythematosus
SS	Sjögren’s syndrome
Teff	Effector T cells
TFH	T follicular helper
TGF	Transforming growth factor
T_H_1	T helper 1
T_H_17	T helper 17
TLR	Toll-like receptor
TNF	Tumor necrosis factor
T_reg_	Regulatory T cells
VEGF	Vascular endothelial growth factor
VKH	Vogt-Koyanagi-Harada

## References

[1] Gill S.R., Pop M., DeBoy R.T., Eckburg P.B., Turnbaugh P.J., Samuel B.S., Gordon J.I., Relman D.A., Fraser-Liggett C.M., Nelson K.E. (2006). Metagenomic analysis of the human distal gut microbiome. Science.

[2] Peterson J., Garges S., Giovanni M., McInnes P., Wang L., Schloss J.A., Bonazzi V., McEwen J.E., Wetterstrand K.A., Deal C. (2009). The NIH Human Microbiome Project. Genome Res..

[3] Integrative H.M.P., Proctor L.M., Creasy H.H., Fettweis J.M., Lloyd-Price J., Mahurkar A., Zhou W., Buck G.A., Snyder M.P., Strauss J.F. (2019). The Integrative Human Microbiome Project. Nat. Cell Biol..

[4] Kahrstrom C.T., Pariente N., Weiss U. (2016). Intestinal microbiota in health and disease. Nature.

[5] Lee Y.K., Mazmanian S.K. (2010). Has the microbiota played a critical role in the evolution of the adaptive immune system?. Science.

[6] Berer K., Mues M., Koutrolos M., Al Rasbi Z., Boziki M., Johner C., Wekerle H., Krishnamoorthy G. (2011). Commensal microbiota and myelin autoantigen cooperate to trigger autoimmune demyelination. Nature.

[7] Fleischmann R.D., Adams M.D., White O., Clayton A.R., Kirkness E.F., Kerlavage A.R., Bult C.J., Tomb J.F., Dougherty B.A., Merrick J.M. (1995). Whole-genome random sequencing and assembly of Haemophilus influenzae Rd. Science.

[8] Relman D.A. (2011). Microbial Genomics and Infectious Diseases. N. Engl. J. Med..

[9] Gentile C.L., Weir T.L. (2018). The gut microbiota at the intersection of diet and human health. Science.

[10] Segre J.A. (2015). Microbial growth dynamics and human disease. Science.

[11] Lynch S.V., Pedersen O. (2016). The Human Intestinal Microbiome in Health and Disease. N. Engl. J. Med..

[12] Both T., Dalm V.A., Van Hagen P.M., van Daele P.L. (2017). Reviewing primary Sjögren’s syndrome: Beyond the dryness—From pathophysiology to diagnosis and treatment. Int. J. Med. Sci..

[13] Kuklinski E., Asbell P.A. (2017). Sjogren’s syndrome from the perspective of ophthalmology. Clin. Immunol..

[14] Jones L., Downie L.E., Korb D., Benitez-Del-Castillo J.M., Dana R., Deng S.X., Dong P.N., Geerling G., Hida R.Y., Liu Y. (2017). TFOS DEWS II Management and Therapy Report. Ocul. Surf..

[15] Craig J.P., Nelson J.D., Azar D.T., Belmonte C., Bron A., Chauhan S.K., De Paiva C.S., Gomes J.A., Hammitt K.M., Jones L.W. (2017). TFOS DEWS II Report Executive Summary. Ocul. Surf..

[16] Horai R., Caspi R.R. (2019). Microbiome and Autoimmune Uveitis. Front. Immunol..

[17] Pascal V., Pozuelo M., Borruel N., Casellas F., Campos D., Santiago A., Martinez X., Varela E., Sarrabayrouse G., Machiels K. (2017). A microbial signature for Crohn’s disease. Gut.

[18] Zárate-Bladés C.R., Horai R., Mattapallil M.J., Ajami N.J., Wong M., Petrosino J.F., Itoh K., Chan C.-C., Caspi R.R. (2017). Gut microbiota as a source of a surrogate antigen that triggers autoimmunity in an immune privileged site. Gut Microbes.

[19] Silverman G.J. (2019). The microbiome in SLE pathogenesis. Nat. Rev. Rheumatol..

[20] Li Z., Zhu H., Zhang L., Qin C. (2018). The intestinal microbiome and Alzheimer’s disease: A review. Anim. Model Exp. Med..

[21] Opazo M.C., Ortega-Rocha E.M., Coronado-Arrázola I., Bonifaz L.C., Boudin H., Neunlist M., Bueno S.M., Kalergis A.M., Riedel C.A. (2018). Intestinal Microbiota Influences Non-intestinal Related Autoimmune Diseases. Front. Microbiol..

[22] Rujillo-Vargas C.M., Schaefer L., Alam J., Pflugfelder S.C., Britton R.A., De Paiva C.S. (2020). The gut-eye-lacrimal gland-microbiome axis in Sjögren Syndrome. Ocul. Surf..

[23] Fabbiano S., Suárez-Zamorano N., Trajkovski M. (2017). Host–Microbiota Mutualism in Metabolic Diseases. Front. Endocrinol..

[24] Depommier C., Everard A., Druart C., Plovier H., Van Hul M., Vieira-Silva S., Falony G., Raes J., Maiter D., Delzenne N.M. (2019). Supplementation with Akkermansia muciniphila in overweight and obese human volunteers: A proof-of-concept exploratory study. Nat. Med..

[25] Muller P.A., Schneeberger M., Matheis F., Wang P., Kerner Z., Ilanges A., Pellegrino K., Del Mármol J., Castro T.B.R., Furuichi M. (2020). Microbiota modulate sympathetic neurons via a gut-brain circuit. Nat. Cell Biol..

[26] Zhao Q., Elson C.O. (2018). Adaptive immune education by gut microbiota antigens. Immunology.

[27] Thaiss C.A., Zmora N., Levy M., Elinav E. (2016). The microbiome and innate immunity. Nat. Cell Biol..

[28] Jiao Y., Wu L., Huntington N.D., Zhang X. (2020). Crosstalk Between Gut Microbiota and Innate Immunity and Its Implication in Autoimmune Diseases. Front. Immunol..

[29] Honda K., Littman D.R. (2016). The microbiota in adaptive immune homeostasis and disease. Nat. Cell Biol..

[30] Zheng Y., Valdez P.A., Danilenko D.M., Hu Y., Sa S.M., Gong Q., Abbas A.R., Modrusan Z., Ghilardi N., De Sauvage F.J. (2008). Interleukin-22 mediates early host defense against attaching and effacing bacterial pathogens. Nat. Med..

[31] Zhang D., Chen G., Manwani D., Mortha A., Xu C., Faith J.J., Burk R.D., Kunisaki Y., Jang J.-E., Scheiermann C. (2015). Neutrophil ageing is regulated by the microbiome. Nat. Cell Biol..

[32] Hill D.A., Siracusa M.C., Abt M.C., Kim B.S., Kobuley D., Kubo M., Kambayashi T., LaRosa D.F., Renner E.D., Orange J.S. (2012). Commensal bacteria—Derived signals regulate basophil hematopoiesis and allergic inflammation. Nat. Med..

[33] Wang L., Zhu L., Qin S. (2019). Gut Microbiota Modulation on Intestinal Mucosal Adaptive Immunity. J. Immunol. Res..

[34] Zheng D., Liwinski T., Elinav E. (2020). Interaction between microbiota and immunity in health and disease. Cell Res..

[35] Guzman-Bautista E.R., Suzuki K., Asami S., Fagarasan S. (2020). Bacteria-immune cells dialog and the homeostasis of the systems. Curr. Opin. Immunol..

[36] van Der Meulen T.A., Harmsen H.J., Vila A.V., Kurilshikov A., Liefers S.C., Zhernakova A., Fu J., Wijmenga C., Weersma R.K., de Leeuw K. (2019). Shared gut, but distinct oral microbiota composition in primary Sjögren’s syndrome and systemic lupus erythematosus. J. Autoimmun..

[37] Wang D., Lei L. (2020). Interleukin-35 regulates the balance of Th17 and Treg responses during the pathogenesis of connective tissue diseases. Int. J. Rheum. Dis..

[38] Jiang J., Zhao M., Chang C., Wu H., Lu Q. (2020). Type I Interferons in the Pathogenesis and Treatment of Autoimmune Diseases. Clin. Rev. Allergy Immunol..

[39] Scher J.U., Sczesnak A., Longman R.S., Segata N., Ubeda C., Bielski C., Rostron T., Cerundolo V., Pamer E.G., Abramson S.B. (2013). Expansion of intestinal Prevotella copri correlates with enhanced susceptibility to arthritis. eLife.

[40] Chen J., Wright K., Davis J.M., Jeraldo P., Marietta E.V., Murray J., Nelson H., Matteson E.L., Taneja V. (2016). An expansion of rare lineage intestinal microbes characterizes rheumatoid arthritis. Genome Med..

[41] Kim J.-W., Kwok S.-K., Choe J.-Y., Park S.-H. (2019). Recent Advances in Our Understanding of the Link between the Intestinal Microbiota and Systemic Lupus Erythematosus. Int. J. Mol. Sci..

[42] Azzouz D., Omarbekova A., Heguy A., Schwudke D., Gisch N., Rovin B.H., Caricchio R., Buyon J.P., Alekseyenko A.V., Silverman G.J. (2019). Lupus nephritis is linked to disease-activity associated expansions and immunity to a gut commensal. Ann. Rheum. Dis..

[43] Guo M., Wang H., Xu S., Zhuang Y., An J., Su C., Xia Y., Chen J., Xu Z.Z., Liu Q. (2020). Alteration in gut microbiota is associated with dysregulation of cytokines and glucocorticoid therapy in systemic lupus erythematosus. Gut Microbes.

[44] Vaahtovuo J., Munukka E., Korkeamäki M., Luukkainen R., Toivanen P. (2008). Fecal microbiota in early rheumatoid arthritis. J. Rheumatol..

[45] He Z., Shao T., Li H., Xie Z., Wen C. (2016). Alterations of the gut microbiome in Chinese patients with systemic lupus erythematosus. Gut Pathog..

[46] Hevia A., Milani C., López P., Cuervo A., Arboleya S., Duranti S., Turroni F., González S., Suárez A., Gueimonde M. (2014). Intestinal Dysbiosis Associated with Systemic Lupus Erythematosus. mBio.

[47] Luo X.M., Edwards M.R., Mu Q., Yu Y., Vieson M.D., Reilly C.M., Ahmed S.A., Bankole A.A. (2017). Gut Microbiota in Human Systemic Lupus Erythematosus and a Mouse Model of Lupus. Appl. Environ. Microbiol..

[48] Zegarra-Ruiz D.F., El Beidaq A., Iñiguez A.J., Di Ricco M.L., Vieira S.M., Ruff W.E., Mubiru D., Fine R.L., Sterpka J., Greiling T.M. (2019). A Diet-Sensitive Commensal Lactobacillus Strain Mediates TLR7-Dependent Systemic Autoimmunity. Cell Host Microbe.

[49] Rudbane S.M.A., Rahmdel S., Abdollahzadeh S.M., Zare M., Bazrafshan A., Mazloomi S.M. (2018). The efficacy of probiotic supplementation in rheumatoid arthritis: A meta-analysis of randomized, controlled trials. Inflammopharmacology.

[50] de la Visitación N., Robles-Vera I., Toral M., Duarte J. (2019). Protective Effects of Probiotic Consumption in Cardiovascular Disease in Systemic Lupus Erythematosus. Nutrients.

[51] Johnson K.V.-A., Foster K.R. (2018). Why does the microbiome affect behaviour?. Nat. Rev. Genet..

[52] Martin C.R., Osadchiy V., Kalani A., Mayer E.A. (2018). The Brain-Gut-Microbiome Axis. Cell Mol. Gastroenterol. Hepatol..

[53] Holzer P., Farzi A. (2014). Neuropeptides and the Microbiota-Gut-Brain Axis. Adv. Exp. Med. Biol..

[54] Hajjo H., Geva-Zatorsky N. (2020). Gut microbiota—Host interactions now also brain-immune axis. Curr. Opin. Neurobiol..

[55] Barbosa R.S.D., Vieira-Coelho M.A. (2019). Probiotics and prebiotics: Focus on psychiatric disorders—A systematic review. Nutr. Rev..

[56] Gong H., Zhang S., Li Q., Zuo C., Gao X., Zheng B., Lin M. (2020). Gut microbiota compositional profile and serum metabolic phenotype in patients with primary open-angle glaucoma. Exp. Eye Res..

[57] Ye Z., Wu C., Zhang N., Du L., Cao Q., Huang X., Tang J., Wang Q., Li F., Zhou C. (2020). Altered gut microbiome composition in patients with Vogt-Koyanagi-Harada disease. Gut Microbes.

[58] Rowan S., Jiang S., Korem T., Szymanski J., Chang M.-L., Szelog J., Cassalman C., Dasuri K., McGuire C., Nagai R. (2017). Involvement of a gut–retina axis in protection against dietary glycemia-induced age-related macular degeneration. Proc. Natl. Acad. Sci. USA.

[59] Horai R., Zarateblades C.R., Dillenburg-Pilla P., Chen J., Kielczewski J.L., Silver P.B., Jittayasothorn Y., Chan C.-C., Yamane H., Honda K. (2015). Microbiota-Dependent Activation of an Autoreactive T Cell Receptor Provokes Autoimmunity in an Immunologically Privileged Site. Immunity.

[60] Huang X., Ye Z., Cao Q., Su G., Wang Q., Deng J., Zhou C., Kijlstra A., Yang P. (2018). Gut Microbiota Composition and Fecal Metabolic Phenotype in Patients With Acute Anterior Uveitis. Investig. Opthalmology Vis. Sci..

[61] Ye Z., Zhang N., Wu C., Zhang X., Wang Q., Huang X., Du L., Cao Q., Tang J., Zhou C. (2018). A metagenomic study of the gut microbiome in Behcet’s disease. Microbiome.

[62] Shimizu J., Kubota T., Takada E., Takai K., Fujiwara N., Arimitsu N., Ueda Y., Wakisaka S., Suzuki T., Suzuki N. (2019). Relative abundance of Megamonas hypermegale and Butyrivibrio species decreased in the intestine and its possible association with the T cell aberration by metabolite alteration in patients with Behcet’s disease (210 characters). Clin. Rheumatol..

[63] Janowitz C., Nakamura Y.K., Metea C., Gligor A., Yu W., Karstens L., Rosenbaum J.T., Asquith M., Lin P. (2019). Disruption of Intestinal Homeostasis and Intestinal Microbiota During Experimental Autoimmune Uveitis. Investig. Opthalmology Vis. Sci..

[64] Du Z., Wang Q., Huang X., Yi S., Mei S., Yuan G., Su G., Cao Q., Zhou C., Wang Y. (2020). Effect of berberine on spleen transcriptome and gut microbiota composition in experimental autoimmune uveitis. Int. Immunopharmacol..

[65] Chakravarthy S.K., Jayasudha R., Prashanthi G.S., Ali M.H., Sharma S., Tyagi M., Shivaji S. (2018). Dysbiosis in the Gut Bacterial Microbiome of Patients with Uveitis, an Inflammatory Disease of the Eye. Indian J. Microbiol..

[66] Zinkernagel M.S., Zysset-Burri D.C., Keller I., Berger L.E., Leichtle A.B., Largiadèr C.R., Fiedler G.M., Wolf S. (2017). Association of the Intestinal Microbiome with the Development of Neovascular Age-Related Macular Degeneration. Sci. Rep..

[67] Shi Z., Qiu Y., Wang J., Fang Y., Zhang Y., Chen H., Du Q., Zhao Z., Yan C., Yang M. (2020). Dysbiosis of gut microbiota in patients with neuromyelitis optica spectrum disorders: A cross sectional study. J. Neuroimmunol..

[68] Gong J., Qiu W., Zeng Q., Liu X., Sun X., Li H., Yang Y., Wu A., Bao J., Wang Y. (2018). Lack of short-chain fatty acids and overgrowth of opportunistic pathogens define dysbiosis of neuromyelitis optica spectrum disorders: A Chinese pilot study. Mult. Scler. J..

[69] Cree B.A.C., Spencer C.M., Varrin-Doyer M., Baranzini S.E., Zamvil S.S. (2016). Gut microbiome analysis in neuromyelitis optica reveals overabundance of Clostridium perfringens. Ann. Neurol..

[70] Lin P., Bach M., Asquith M., Lee A.Y., Akileswaran L., Stauffer P., Davin S., Pan Y., Cambronne E.D., Dorris M. (2014). HLA-B27 and Human beta2-Microglobulin Affect the Gut Microbiota of Transgenic Rats. PLoS ONE.

[71] Andriessen E.M., Wilson A.M., Mawambo G., Dejda A., Miloudi K., Sennlaub F., Sapieha P. (2016). Gut microbiota influences pathological angiogenesis in obesity-driven choroidal neovascularization. EMBO Mol. Med..

[72] Kugadas A., Wright Q., Geddes-McAlister J., Gadjeva M. (2017). Role of Microbiota in Strengthening Ocular Mucosal Barrier Function Through Secretory IgA. Investig. Opthalmology Vis. Sci..

[73] Kodati S., Sen H.N. (2019). Uveitis and the gut microbiota. Best Pract. Res. Clin. Rheumatol..

[74] Horai R., Sen H.N., Caspi R.R. (2017). Commensal microbiota as a potential trigger of autoimmune uveitis. Expert Rev. Clin. Immunol..

[75] Nakamura Y.K., Metea C., Karstens L., Asquith M., Gruner H., Moscibrocki C., Lee I., Brislawn C.J., Jansson J.K., Rosenbaum J.T. (2016). Gut Microbial Alterations Associated With Protection From Autoimmune Uveitis. Investig. Opthalmol. Vis. Sci..

[76] Du L., Kijlstra A., Yang P. (2016). Vogt-Koyanagi-Harada disease: Novel insights into pathophysiology, diagnosis and treatment. Prog. Retin. Eye Res..

[77] Liang L., Tan X., Zhou Q., Tian Y., Kijlstra A., Yang P. (2015). TLR3 and TLR4 But not TLR2 are Involved in Vogt-Koyanagi-Harada Disease by Triggering Proinflammatory Cytokines Production Through Promoting the Production of Mitochondrial Reactive Oxygen Species. Curr. Mol. Med..

[78] Rinninella E., Mele M.C., Merendino N., Cintoni M., Anselmi G., Caporossi A., Gasbarrini A., Minnella A.M. (2018). The Role of Diet, Micronutrients and the Gut Microbiota in Age-Related Macular Degeneration: New Perspectives from the Gut–Retina Axis. Nutrients.

[79] Lin P. (2019). Importance of the intestinal microbiota in ocular inflammatory diseases: A review. Clin. Exp. Ophthalmology.

[80] Gill T., Asquith M., Rosenbaum J.T., Colbert R.A. (2015). The intestinal microbiome in spondyloarthritis. Curr. Opin. Rheumatol..

[81] Mancino R., Martucci A., Cesareo M., Giannini C., Corasaniti M.T., Bagetta G., Nucci C. (2018). Glaucoma and Alzheimer Disease: One Age-Related Neurodegenerative Disease of the Brain. Curr. Neuropharmacol..

[82] Skrzypecki J., Żera T., Ufnal M. (2018). Butyrate, a Gut Bacterial Metabolite, Lowers Intraocular Pressure in Normotensive But Not in Hypertensive Rats. J. Glaucoma.

[83] Ratelade J., Verkman A. (2012). Neuromyelitis optica: Aquaporin-4 based pathogenesis mechanisms and new therapies. Int. J. Biochem. Cell Biol..

[84] Wang C., Zaheer M., Bian F., Quach D., Swennes A.G., Britton R.A., Pflugfelder S.C., De Paiva C.S. (2018). Sjögren-Like Lacrimal Keratoconjunctivitis in Germ-Free Mice. Int. J. Mol. Sci..

[85] Yanagisawa N., Ueshiba H., Abe Y., Kato H., Higuchi T., Yagi J. (2018). Outer Membrane Protein of Gut Commensal Microorganism Induces Autoantibody Production and Extra-Intestinal Gland Inflammation in Mice. Int. J. Mol. Sci..

[86] Choi S.H., Oh J.W., Ryu J.S., Kim H.M., Im S.-H., Kim K.P., Kim M.K. (2020). IRT5 Probiotics Changes Immune Modulatory Protein Expression in the Extraorbital Lacrimal Glands of an Autoimmune Dry Eye Mouse Model. Investig. Opthalmology Vis. Sci..

[87] Bron A.J., De Paiva C.S., Chauhan S.K., Bonini S., Gabison E.E., Jain S., Knop E., Markoulli M., Ogawa Y., Perez V. (2017). TFOS DEWS II pathophysiology report. Ocul. Surf..

[88] Chauhan S.K., Dana R. (2009). Role of Th17 cells in the immunopathogenesis of dry eye disease. Mucosal Immunol..

[89] de Paiva C.S., Chotikavanich S., Pangelinan S.B., Pitcher J.D., Fang B., Zheng X., Ma P., Farley W.J., Siemasko K.F., Niederkorn J.Y. (2009). IL-17 disrupts corneal barrier following desiccating stress. Mucosal Immunol..

[90] Albert L.J., Inman R.D. (1999). Molecular Mimicry and Autoimmunity. N. Engl. J. Med..

[91] Ivanov I.I., Atarashi K., Manel N., Brodie E.L., Shima T., Karaoz U., Wei D., Goldfarb K.C., Santee C.A., Lynch S.V. (2009). Induction of Intestinal Th17 Cells by Segmented Filamentous Bacteria. Cell.

[92] Stern M.E., Schaumburg C.S., Pflugfelder S.C. (2013). Dry Eye as a Mucosal Autoimmune Disease. Int. Rev. Immunol..

[93] Perez V.L., Pflugfelder S.C., Zhang S., Shojaei A., Haque R. (2016). Lifitegrast, a Novel Integrin Antagonist for Treatment of Dry Eye Disease. Ocul. Surf..

[94] Yao Y., Ma J.-F., Chang C., Xu T., Gao C.-Y., Gershwin M.E., Lian Z.-X. (2020). Immunobiology of T Cells in Sjögren’s Syndrome. Clin. Rev. Allergy Immunol..

[95] Ainola M., Porola P., Takakubo Y., Przybyla B., Kouri V.P., Tolvanen T.A., Hänninen A., Nordström D.C. (2018). Activation of plasmacytoid dendritic cells by apoptotic particles—Mechanism for the loss of immunological tolerance in Sjögren’s syndrome. Clin. Exp. Immunol..

[96] Verstappen G.M., Corneth O.B., Bootsma H., Kroese F.G. (2018). Th17 cells in primary Sjögren’s syndrome: Pathogenicity and plasticity. J. Autoimmun..

[97] Bacman S., Sterin-Borda L., Camusso J.J., Arana R., Hübscher O., Borda E. (1996). Circulating antibodies against rat parotid gland M3 muscarinic receptors in primary Sjögren’s syndrome. Clin. Exp. Immunol..

[98] Baudouin C., Messmer E.M., Aragona P., Geerling G., Akova Y.A., Benítez-Del-Castillo J., Boboridis K.G., Merayo-Lloves J., Rolando M., Labetoulle M. (2016). Revisiting the vicious circle of dry eye disease: A focus on the pathophysiology of meibomian gland dysfunction. Br. J. Ophthalmol..

[99] Yoon C.H., Ryu J.S., Hwang H.S., Kim M.K. (2020). Comparative Analysis of Age-Related Changes in Lacrimal Glands and Meibomian Glands of a C57BL/6 Male Mouse Model. Int. J. Mol. Sci..

[100] Dogru M., Kojima T., Şimşek C., Tsubota K. (2018). Potential Role of Oxidative Stress in Ocular Surface Inflammation and Dry Eye Disease. Investig. Opthalmology Vis. Sci..

[101] Uchino Y., Kawakita T., Miyazawa M., Ishii T., Onouchi H., Yasuda K., Ogawa Y., Shimmura S., Ishii N., Tsubota K. (2012). Oxidative Stress Induced Inflammation Initiates Functional Decline of Tear Production. PLoS ONE.

[102] Jester J.V., Parfitt G.J., Brown D.J. (2015). Meibomian gland dysfunction: Hyperkeratinization or atrophy?. BMC Ophthalmol..

[103] Ding C., Nandoskar P., Lu M., Thomas P., Trousdale M.D., Wang Y. (2011). Changes of Aquaporins in the Lacrimal Glands of a Rabbit Model of Sjögren’s Syndrome. Curr. Eye Res..

[104] Delporte C., Beitz E. (2009). Aquaporins in Secretory Glands and their Role in Sjögren’s Syndrome. Aquaporins.

[105] Soyfoo M.S., Chivasso C., Perret J., Delporte C. (2018). Involvement of Aquaporins in the Pathogenesis, Diagnosis and Treatment of Sjögren’s Syndrome. Int. J. Mol. Sci..

[106] van Deursen J.M. (2014). The role of senescent cells in ageing. Nat. Cell Biol..

[107] Atarashi K., Tanoue T., Oshima K., Suda W., Nagano Y., Nishikawa H., Fukuda S., Saito T., Narushima S., Hase K. (2013). Treg induction by a rationally selected mixture of Clostridia strains from the human microbiota. Nat. Cell Biol..

[108] Round J.L., Mazmanian S.K. (2010). Inducible Foxp3+ regulatory T-cell development by a commensal bacterium of the intestinal microbiota. Proc. Natl. Acad. Sci. USA.

[109] Smith P.M., Howitt M.R., Panikov N., Michaud M., Gallini C.A., Bohlooly Y.M., Glickman J.N., Garrett W.S. (2013). The Microbial Metabolites, Short-Chain Fatty Acids, Regulate Colonic Treg Cell Homeostasis. Science.

[110] Barr J.Y., Wang X., Meyerholz D.K., Lieberman S.M. (2017). CD8 T cells contribute to lacrimal gland pathology in the nonobese diabetic mouse model of Sjögren syndrome. Immunol. Cell Biol..

[111] Brkic Z., Corneth O., van Helden-Meeuwsen C.G., Dolhain R.J., Maria N.I., Paulissen S.M.J., Davelaar N., Van Hamburg J.P., van Daele P.L., Dalm V.A. (2014). T-helper 17 cell cytokines and interferon type I: Partners in crime in systemic lupus erythematosus?. Arthritis Res. Ther..

[112] Pernis A.B. (2009). Th17 cells in rheumatoid arthritis and systemic lupus erythematosus. J. Intern. Med..

[113] de Paiva C.S., Jones D.B., Stern M.E., Bian F., Moore Q.L., Corbiere S., Streckfus C.F., Hutchinson D.S., Ajami N.J., Petrosino J.F. (2016). Altered Mucosal Microbiome Diversity and Disease Severity in Sjögren Syndrome. Sci. Rep..

[114] Wang C., Schaefer L., Bian F., Yu Z., Pflugfelder S.C., Britton R.A., De Paiva C.S. (2019). Dysbiosis Modulates Ocular Surface Inflammatory Response to Liposaccharide. Investig. Opthalmology Vis. Sci..

[115] Zaheer M., Wang C., Bian F., Yu Z., Hernandez H., De Souza R.G., Simmons K.T., Schady D., Swennes A.G., Pflugfelder S.C. (2018). Protective role of commensal bacteria in Sjögren Syndrome. J. Autoimmun..

[116] Wu Y., Wu J., Bu J., Tang L.-Y., Yang Y., Ouyang W., Lin X., Liu Z., Huang C., Quantock A.J. (2020). High-fat diet induces dry eye-like ocular surface damages in murine. Ocul. Surf..

[117] Szymula A., Rosenthal J., Szczerba B.M., Bagavant H., Fu S.M., Deshmukh U.S. (2014). T cell epitope mimicry between Sjogren’s syndrome Antigen A (SSA)/Ro60 and oral, gut, skin and vaginal bacteria. Clin. Immunol..

[118] Moon J., Choi S.H., Yoon C.H., Kim M.K. (2020). Gut dysbiosis is prevailing in Sjögren’s syndrome and is related to dry eye severity. PLoS ONE.

[119] Mendez R., Watane A., Farhangi M., Cavuoto K.M., Leith T., Budree S., Galor A., Banerjee S. (2020). Gut microbial dysbiosis in individuals with Sjögren’s syndrome. Microb. Cell Factories.

[120] Mandl T., Marsal J., Olsson P., Ohlsson B., Andréasson K. (2017). Severe intestinal dysbiosis is prevalent in primary Sjögren’s syndrome and is associated with systemic disease activity. Arthritis Res..

[121] Hill C., Guarner F., Reid G., Gibson G.R., Merenstein D.J., Pot B., Morelli L., Canani R.B., Flint H.J., Salminen S. (2014). The International Scientific Association for Probiotics and Prebiotics consensus statement on the scope and appropriate use of the term probiotic. Nat. Rev. Gastroenterol. Hepatol..

[122] Gibson G.R., Hutkins R., Sanders M.E., Prescott S.L., Reimer R.A., Salminen S.J., Scott K., Stanton C., Swanson K.S., Cani P.D. (2017). Expert consensus document: The International Scientific Association for Probiotics and Prebiotics (ISAPP) consensus statement on the definition and scope of prebiotics. Nat. Rev. Gastroenterol. Hepatol..

[123] Tsai Y.-L., Lin T.-L., Chang C.-J., Wu T.-R., Lai W.-F., Lu C.-C., Lai H.-C. (2019). Probiotics, prebiotics and amelioration of diseases. J. Biomed. Sci..

[124] Liu Y., Alookaran J.J., Rhoads J.M. (2018). Probiotics in Autoimmune and Inflammatory Disorders. Nutrients.

[125] Kawashima M., Nakamura S., Izuta Y., Inoue S., Tsubota K. (2016). Dietary Supplementation with a Combination of Lactoferrin, Fish Oil, and Enterococcus faecium WB2000 for Treating Dry Eye: A Rat Model and Human Clinical Study. Ocul. Surf..

[126] Kim J., Choi S.H., Kim Y.J., Jeong H.J., Ryu J.S., Lee H.J., Kim T.W., Im S.-H., Oh J.Y., Kim M.K. (2017). Clinical Effect of IRT-5 Probiotics on Immune Modulation of Autoimmunity or Alloimmunity in the Eye. Nutrients.

[127] Chisari G., Chisari E.M., Ozyalcin E., Borzì A.M., Chisari C.G. (2018). Aging Eye Microbiota in Dry Eye Syndrome in Patients Treated with Enterococcus faecium and Saccharomyces boulardii. Curr. Clin. Pharmacol..

[128] Chisari G., Chisari E.M., Francaviglia A., Chisari C.G. (2017). The mixture of bifidobacterium associated with fructo-oligosaccharides reduces the damage of the ocular surface. La Clin. Ter..

[129] Kawashima M., Tsuno S., Matsumoto M., Tsubota K. (2019). Hydrogen-producing milk to prevent reduction in tear stability in persons using visual display terminals. Ocul. Surf..

[130] Hansen C.H.F., Larsen C.S., Petersson H.O., Zachariassen L.F., Vegge A., Lauridsen C., Kot W., Krych Ł., Nielsen D.S., Hansen A.K. (2019). Targeting gut microbiota and barrier function with prebiotics to alleviate autoimmune manifestations in NOD mice. Diabetologia.

[131] Ghattargi V., Gaikwad M.A., Meti B.S., Nimonkar Y.S., Dixit K., Prakash O., Shouche Y.S., Pawar S.P., Dhotre D. (2018). Comparative genome analysis reveals key genetic factors associated with probiotic property in Enterococcus faecium strains. BMC Genom..

[132] Azad M., Kalam A., Sarker M., Li T., Yin J. (2018). Probiotic Species in the Modulation of Gut Microbiota: An Overview. Biomed Res. Int..

[133] Papizadeh M., Rohani M., Nahrevanian H., Javadi A., Pourshafie M.R. (2017). Probiotic characters of Bifidobacterium and Lactobacillus are a result of the ongoing gene acquisition and genome minimization evolutionary trends. Microb. Pathog..

[134] MacGregor G., Smith A.J., Thakker B., Kinsella J. (2002). Yoghurt biotherapy: Contraindicated in immunosuppressed patients?. Postgrad. Med. J..

[135] Pflugfelder S.C., Corrales R.M., De Paiva C.S. (2013). T helper cytokines in dry eye disease. Exp. Eye Res..

[136] Clemente J.C., Manasson J., Scher J.U. (2018). The role of the gut microbiome in systemic inflammatory disease. BMJ.

[137] Asano Y., Hiramoto T., Nishino R., Aiba Y., Kimura T., Yoshihara K., Koga Y., Sudo N. (2012). Critical role of gut microbiota in the production of biologically active, free catecholamines in the gut lumen of mice. Am. J. Physiol. Liver Physiol..

